# Uterine fluid proteome changes during diapause and resumption of embryo development in roe deer (*Capreolus capreolus*)

**DOI:** 10.1530/REP-19-0022

**Published:** 2019-04-01

**Authors:** V A van der Weijden, J T Bick, S Bauersachs, G J Arnold, T Fröhlich, B Drews, S E Ulbrich

**Affiliations:** 1 ETH Zurich, Animal Physiology, Institute of Agricultural Sciences, Zurich, Switzerland; 2 Genetics and Functional Genomics, Clinic of Reproductive Medicine, Department for Farm Animals, University of Zurich, Zurich, Switzerland; 3 Laboratory for Functional Genome Analysis LAFUGA, Ludwig-Maximilians-Universität Munich, Munich, Germany

## Abstract

The uterine microenvironment during pre-implantation presents a pro-survival milieu and is essential for embryo elongation in ruminants. The European roe deer (*Careolus capreolus*) pre-implantation embryo development is characterised by a 4-month period of reduced development, embryonic diapause, after which the embryo rapidly elongates and implants. We investigated the uterine fluid proteome by label-free liquid chromatography tandem mass spectrometry at four defined stages covering the phase of reduced developmental pace (early diapause, mid-diapause and late diapause) and embryo elongation. We hypothesised that embryo development during diapause is halted by the lack of signals that support progression past the blastocyst stage. Three clusters of differentially abundant proteins were identified by a self-organising tree algorithm: (1) gradual reduction over development; (2) stable abundance during diapause, followed by a sharp rise at elongation; and (3) gradual increase over development. Proteins in the different clusters were subjected to gene ontology analysis. ‘Cellular detoxification’ in cluster 1 was represented by alcohol dehydrogenase, glutathione S-transferase and peroxiredoxin-2. ATP-citrate synthase, nucleolin, lamin A/C, and purine phosphorylase as cell proliferation regulators were found in cluster 2 and ‘cortical cytoskeleton’, ‘regulation of substrate adhesion-dependent cell spreading’ and ‘melanosome’ were present in cluster 3. Cell cycle promoters were higher abundant at elongation than during diapause, and polyamines presence indicates their role in diapause regulation. This study provides a comprehensive overview of proteins in the roe deer uterine fluid during diapause and forms a basis for studies aiming at understanding the impact of the lack of cell cycle promoters during diapause.

## Introduction

The uterine environment changes constantly during early embryo development to support survival and the establishment of pregnancy. Until the blastocyst stage, the embryo develops relatively autonomous and independently of the milieu of the maternal tract (Munoz *et al.* 2012). Its adopting to different culture conditions with a relatively high degree of flexibility substantiates this. Once the embryo hatches, proteins in the uterine fluid are essential for embryo development past the blastocyst stage. A number of proteins are hypothesised to support embryo elongation (Bazer 1975), which has to date not been shown to occur *in vitro*. By employing the uterine gland-knockout sheep model, Gray *et al.* confirmed that glandular secretions are a prerequisite for embryo elongation and establishment of pregnancy (Gray *et al.* 2001, 2002). The composition of the uterine fluid presents a mixture of embryonic and endometrial secretions. Morphological changes of the embryo coincide with changes of the uterine fluid composition such as proteins, amino acids, lipids and ions (Forde *et al.* 2014).

Embryonic diapause describes a reproductive strategy in which fertilisation and subsequent embryo implantation are decoupled. In species displaying diapause, the uterine environment supports embryo survival for a prolonged period of time without negatively affecting embryo survival (Renfree & Fenelon 2017). It has been suggested that the uterine secretions supply sufficient nutrients for the embryo to maintain it in a viable state during diapause, but might lack stimulatory factors to initiate resumption of embryonic development (Mead 1993).

The European roe deer (*Capreolus capreolus*) is one of the species in which embryonic diapause is characterised by very slow intrauterine growth at the hatched blastocyst stage (Renfree & Fenelon 2017). A substantial number of studies explored diapause in roe deer, but until now, the key signals causing entry of diapause and reactivation of embryo development have not been described. In 1854, Bischoff suggested that the reproductive tract of the mother had to be the primary cause of diapause (Bischoff 1854). His large observational study showed that the roe deer buck provides functional semen in June, July and August, and that rut, copulation and fertilisation take place between end of July and end of August (Bischoff 1854). The fertilised egg stays in the oviduct until mid-August and rests in the uterus from mid-August until the end of December (Bischoff 1854). After diapause, roe deer embryonic development resembles that of other ruminants (Bischoff 1854, Keibel 1902). Like in other ruminants, embryo elongation has been associated with the release of secretions from the endometrial glands (Aitken *et al.* 1973, Lambert 1999). These secretions were found to be up to 1.5-fold increased during implantation compared to diapause (Aitken *et al.* 1973, Lambert 1999). A first exploratory analysis of the biochemical nature of the uterine fluid during elongation showed a rise in hexose, fructose, total protein, α-amino nitrogen and calcium concentration (Aitken 1974*a*,*b*, 1976). However, there was no association between elongation and secretion of zinc, glucose and prostaglandin F_2α_ (Aitken 1974*a*,*b*). A more recent study from Lambert (2005) identified a specific secreted protein from the roe deer trophoblast cells, which has the same molecular weight and isoelectric point (pI) as pregnancy-associated glycoproteins (PAGs) (Lambert 2005). The abundance of PAG, which Lambert found elevated in maternal serum after implantation, was hypothesised to induce a rapid increase in maternal serum oestradiol concentrations, which has been linked to endometrial preparation for implantation (Lambert 2005). To our knowledge, no further studies have been conducted to examine the composition of the roe deer uterine fluid. Other mammalian species that undergo diapause show a distinct presence of factors implicated in the regulation of diapause such as leukaemia-inhibiting factor (LIF), insulin-like growth factors (IGF), epidermal growth factor (EGF), platelet-derived growth factor (PDGF), fibroblast growth factor (FGF) and transforming growth factor beta (TGFβ) (Renfree & Shaw 2000). In the European mink, the inhibition of ornithine decarboxylase 1 (ODC1), an enzyme involved in the synthesis of polyamines, has been demonstrated to induce diapause and an increased synthesis of polyamines has been shown to reactivate embryo development (Lefevre *et al.* 2011). Specifically, it has been proven that polyamine deprivation results in mitotic arrest in embryos (Lefevre *et al.* 2011) and that hormonal stimulation can regulate the expression of *ODC1* via *pSTAT1* and *mTOR* (Fenelon *et al.* 2016). More recently, and in line with the importance of polyamines, embryonic MYC depletion and mTOR inhibition have been confirmed to induce diapause in mice (Bulut-Karslioglu *et al.* 2016, Scognamiglio *et al.* 2016).

Collectively, this emphasises the importance of proteins for embryo survival and indicates a possible role in controlling embryonic diapause. We hypothesise that the roe deer uterine fluid is a pro-survival environment that halts embryo development during the prolonged period of diapause between September and December and that it lacks signals to support embryonic progression past the blastocyst stage. We analysed the protein abundance at four defined developmental stages covering the period of diapause and embryo elongation to gain insight into prolonged survival during embryonic diapause and its regulation.

## Methods

### Sample collection

The reproductive tract of 225 female roe deer was obtained during regular huntings from September 2015 to January 2016 and September 2016 to January 2017. Ethical approval was neither required nor available as field sampling was performed over the course of regular huntings, where no animals were killed for research purposes. After opening the carcass at the gathering place, the samples were kept on ice until flushing. The average time elapsed between death and uterus removal was 3 h, with a maximum of 4 h. Samples were generally obtained within 1 h after uterus removal. The uterus was freed from connective tissue and flushed with a volume of 2.5 mL phosphate buffered saline (PBS). The embryos were visualised under a stereo microscope (SteREO Discovery Microscope V8, 1:8 Zoom rate, Zeiss), and pictures were taken with the Olympus SC50 camera. The embryo diameter was measured and the embryos were snap frozen in liquid N_2_ after washing them twice in fresh PBS. The uterine fluid was centrifuged for 10 min at 4°C at 800 × ***g*** to pellet any remaining cells. The supernatant was then snap frozen and stored at −80°C until further analysis.

### Isolation of embryonic nucleic acids and experimental groups

Embryonic DNA and total RNA were extracted with the Qiagen AllPrep DNA/RNA micro kit (Qiagen, cat.# 80284) according to manufacturers’ instructions. The genomic DNA content was determined with the Promega Quantus and the QuantiFluor® ONE dsDNA System (Promega, cat.# E4870) according to manufacturers’ instructions. Total RNA was stored at −80°C for further analyses. The total genomic DNA content was calculated and used for the estimation of the total number of cells as a proxy for the developmental stage of each embryo. Four groups were defined based on the embryonic genomic DNA content and morphological characteristics (blastocyst or elongated), and *n* = 5 animals/group were randomly assigned as representative samples for analysis. The four groups referred to as early, mid and late diapause and elongated embryos had a mean ± s.e.m. number of cells of 951 ± 63, 2254 ± 70, 3670 ± 171 and 325,132 ± 40,194, respectively. Early diapause samples were collected between October 21^st^ and November 12^th^, mid-diapause samples were collected between November 21^st^ and December 3^th^, late diapause samples were collected between December 5^th^ and December 28^th^, and elongated samples were collected between November 25^th^ and January 6^th^.

### Quantification of total protein content

Quantification of total protein content in uterine flushing was performed with the Pierce™ BCA Protein Assay Kit (ThermoScientific, prod # 23225) according to manufacturers’ instructions. The standard curve had a range from 0 to 2000 µg/mL BSA. Ten microliter standard and 10 µL undiluted sample were mixed with 200 µL of 50:1 reagent A:B. The plate was shaken for 10 s, followed by 30 min of incubation at 37°C, after which the absorbance was measured at 540 nm.

### Generation of the roe deer proteome database

Data processing was performed on our locally installed Galaxy system (version 18.09) (Blankenberg *et al.* 2010). The protein sequences were obtained by using TransDecoder (Haas *et al.* 2013, Douglas 2018) with a minimum protein length of 100 amino acids. Identified proteins were annotated using BLASTp (Altschul *et al.* 1990, Cock *et al.* 2015) against the human (GRCh38.p12) and bovine (Bos_taurus_UMD_3.1.1) proteome available at NCBI (ftp://ftp.ncbi.nlm.nih.gov/genomes/Homo_sapiens/protein/protein.fa.gz, ftp://ftp.ncbi.nlm.nih.gov/genomes/Bos_taurus/ARCHIVE/ANNOTATION_RELEASE.105/protein/protein.fa.gz).

### LC-MS/MS analysis for protein identification and quantification

Twenty-five micrograms of total protein diluted in 45 µL 50 mM NH_4_HCO_3_ were reduced using dithioerythritol (DTE) at a final concentration of 5 mM for 30 min at 37°C. Cysteins were blocked using iodoacetamide (final concentration 15 mM) at room temperature for 30 min in the dark. Proteins were first digested for 4 h at 37°C using 250 ng LysC (FUJIFILM Wako Pure Chemicals, Osaka, Japan) and then overnight at 37°C with 500 ng porcine trypsin (Promega). Five micrograms of peptides diluted in 0.1% formic acid (FA) were injected into an Ultimate 3000 (Thermo Scientific) nano-chromatography system and transferred to a trap column (PepMap 100 C18, 100 µm × 2 cm, 5 µM particles, Thermo Scientific) at a flow rate of 20 µL/min of solvent A (0.1% FA). Peptides were separated at 250 nL/min (column: PepMap RSLC C18, 75 µm × 50 cm, 2 µm particles, Thermo Scientific) with a 160 min gradient from 5% solvent A to 25% solvent B (0.1% FA in acetonitrile) and a subsequent 10 min gradient from 25 to 40% solvent B. For MS acquisition a Q Exactive HFX (Thermo Scientific) instrument and a top 15 data-dependent method was used. Ion spray voltage was set to 2.2 kV and MS spectra were acquired at a resolution 60,000 (mass-range: 350–1600). The resolution for MS/MS was set to 15,000.

### Data analysis and statistics

The uterine fluid protein concentrations were compared by a one-way ANOVA with Bonferroni *post hoc* in IBM® SPSS® statistics version 23. Acquired raw proteome files were used for protein identification with the MaxQuant Software by matching the ion spectra against the roe deer proteome. Perseus (Tyanova *et al.* 2016) was used for subsequent statistical analysis. In brief, the label-free quantification (LFQ) values were log_2_ transformed and rows were filtered based on valid values, i.e., at least three out of five samples per group in at least one group had valid LFQ values. Subsequently, the imputation feature of Perseus was used to handle missing values. Statistically significant differences in relative abundance of proteins were assessed by a Student’s *t*-test, where different developmental stages were compared. Proteins were considered differentially abundant if the Benjamini–Hochberg adjusted *P* value was ≤0.05 (q value) and the fold-change was ≥1.5. A principle component analysis (PCA), protein abundance visualisation and self-organising tree algorithm (SOTA) were performed in R with prcomp (Wickham 2016), UpSet (Lex *et al.* 2014) and clValid (Brock *et al.* 2008), respectively. Gene ontology (GO) (Ashburner *et al.* 2000) and pathway analysis was conducted using the Database for Annotation, Visualization, and Integrated Discovery (DAVID) with the standard parameter set (Dennis *et al.* 2003). Functional networks integrating the GO-Biological Process, GO-Cellular Component and GO-Molecular Function were graphically displayed using the ClueGO plug-in (Bindea *et al.* 2009, 2013) in Cytoscape 3.6.1 (Shannon *et al.* 2003). A list of all identified proteins including LFQ values can be found in Supplementary Table 1 (see section on supplementary data given at the end of this article).

## Results

### Protein quantification

The uterine fluid protein concentration was not statistically significantly different between the four defined developmental stages. Mean ± s.e.m. protein concentrations for early, mid and late diapause and elongated were 1495 ± 342, 1694 ± 386, 1323 ± 116 and 2039 ± 613 µg/mL, respectively.

### Distinctive developmental stage protein abundancies

A total of 819 proteins were identified and quantified with a false discovery rate (FDR) of <1%. The mean ± s.e.m. number of quantified proteins was 733 ± 39, 589 ± 49, 534 ± 75 and 637 ± 66 at early, mid, late diapause and elongation. The most abundant protein was serum albumin precursor. A total of 107 differentially abundant proteins (DAP) was identified between either one of the six comparisons, while the remaining 712 proteins remained stable irrespective of embryonic developmental stage.

**Figure 1:**
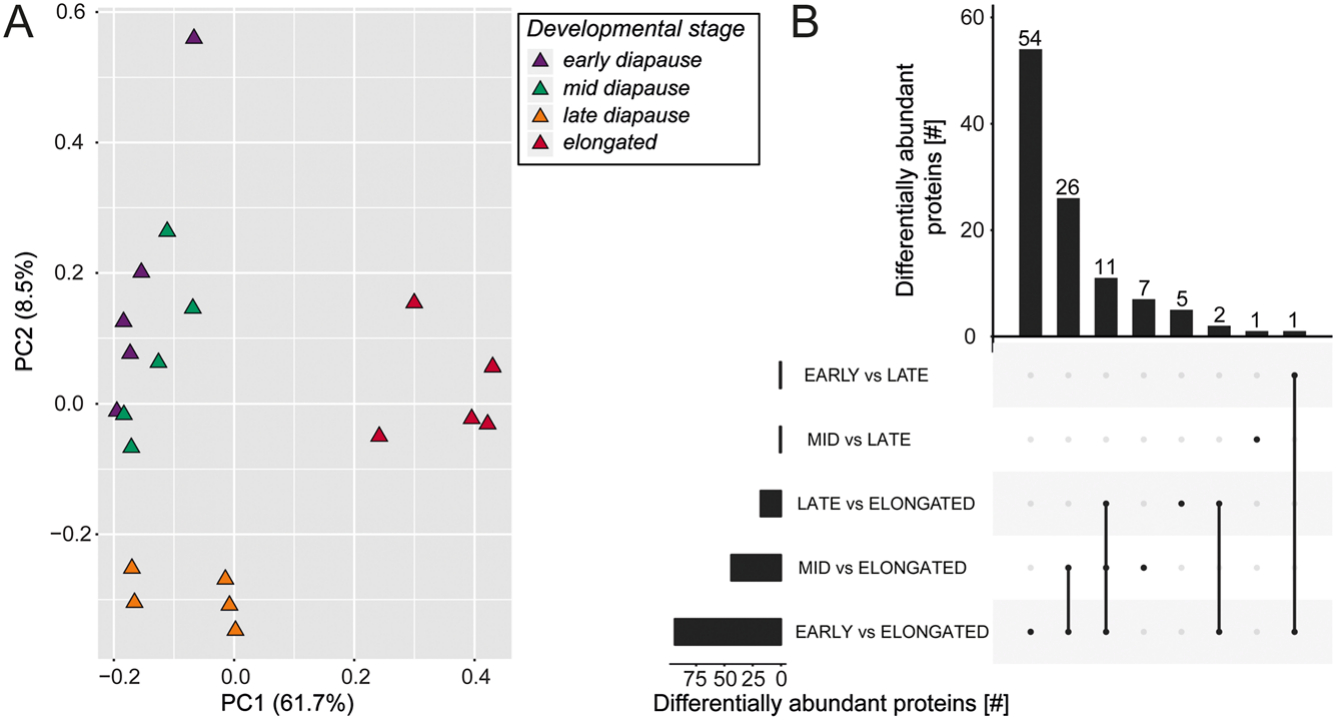
(A) Principle component analysis of differentially abundant proteins in the roe deer uterine fluid. Principle components 1 and 2 explain 61.7 and 8.5% of the variation, respectively. (B) Distribution of the number of DAP between different developmental stages. The dots indicate the DAP for a given comparison, and dots connected with lines indicate that the developmental stage comparisons share this number of DAP.

A PCA of the DAP showed clustering of the early and mid-diapause, and of two separate clusters representing the late diapause and elongated protein profile (Fig. 1A). Most differentially abundant proteins were identified in the uterine fluid comparing early diapause versus elongated embryos (94 DAP, 54 uniquely for early vs elongated), followed by mid-diapause vs elongated (44 DAP, 7 uniquely for mid vs elongated), late diapause vs elongated (18 DAP, 5 uniquely for late vs elongated) and early vs late (1 DAP) and mid vs late diapause (1 uniquely DAP) (Fig. 1B). The single DAP between early and late diapause was IST1 homolog isoform X1, and the single DAP between mid and late diapause was dynactin subunit 3. A full list of DAP with log2 fold-changes is show in Table 1.

**Table 1:** Protein list of differentially abundant proteins with bovine RefSeq protein accession number, proteins name, log2 fold-change (FC) and q-value.

Developmental stage	Protein ID	Protein name	log_2_ FC	q-value
Early – Late Diapause	XP_005218560.1	IST1 homolog isoform X1	2.98	0.022
Mid – Late Diapause	NP_001069128.1	Dynactin subunit 3	−1.90	0.005
Late Diapause – Elongated	NP_001015600.1	Keratin, type I cytoskeletal 19	−5.70	0.0375
	NP_776393.1	Thioredoxin	−5.59	0.0003
	NP_001068616.1	78 kDa glucose-regulated protein precursor	−5.28	0.0140
	XP_002690791.3	Purine nucleoside phosphorylase	−4.64	0.0205
	XP_015331087.1	Ig heavy chain Mem5-like	−4.57	0.0121
	NP_776771.1	10 kDa heat shock protein, mitochondrial	−4.41	0.0139
	XP_005225065.1	Malate dehydrogenase, mitochondrial isoform X1	−4.38	0.0207
	NP_001039876.1	Keratin, type II cytoskeletal 7	−4.38	0.0179
	NP_001179024.1	Keratin, type I cytoskeletal 18	−4.37	0.0403
	NP_001019738.1	Cystathionine gamma-lyase	−4.06	0.0136
	NP_001193589.1	Nucleolin	−3.33	0.0421
	NP_001035610.1	60S ribosomal protein L7a	−2.87	0.0205
	XP_005219340.1	Protein arginine N-methyltransferase 1 isoform X1	−1.69	0.0116
	NP_001028795.1	Phosphatidylethanolamine-binding protein 1	0.87	0.0381
	XP_010814868.1	Growth factor receptor-bound protein 2 isoform X1	1.36	0.0218
	NP_777040.1	Superoxide dismutase (Cu-Zn)	1.40	0.0403
	NP_776917.2	14-3-3 protein eta	1.85	0.0222
	NP_001091040.1	Biliverdin reductase A	2.33	0.0413
Mid-Diapause – Elongated	NP_001028782.1	Keratin, type II cytoskeletal 8	−5.41	0.0339
	NP_001068616.1	78 kDa glucose-regulated protein precursor	−5.16	0.0113
	XP_002690791.3	Purine nucleoside phosphorylase	−5.05	0.0113
	NP_776771.1	10 kDa heat shock protein, mitochondrial	−4.91	0.0070
	NP_001179024.1	Keratin, type I cytoskeletal 18	−4.82	0.0073
	XP_005225065.1	Malate dehydrogenase, mitochondrial isoform X1	−4.52	0.0141
	NP_001193589.1	Nucleolin	−4.33	0.0042
	XP_010819218.1	Protein SET-like	−4.26	0.0087
	XP_015313986.1	60 kDa heat shock protein, mitochondrial isoform X1	−4.12	0.0477
	NP_001019738.1	Cystathionine gamma-lyase	−3.89	0.0087
	XP_002687260.2	Early endosome antigen 1 isoform X2	−3.87	0.0109
	NP_001178987.1	Annexin A10	−3.69	0.0181
	NP_777125.1	Endoplasmin precursor	−3.12	0.0307
	XP_005220772.1	ATP-citrate synthase isoform X2	−2.97	0.0324
	NP_776993.1	Polyadenylate-binding protein 1	−2.72	0.0069
	XP_005211893.1	Kinectin isoform X5	−2.48	0.0322
	NP_001039500.1	Tissue alpha-L-fucosidase precursor	−2.37	0.0391
	NP_786984.1	Isocitrate dehydrogenase (NADP), mitochondrial precursor	−2.22	0.0197
	XP_005219340.1	Protein arginine N-methyltransferase 1 isoform X1	−2.06	0.0081
	NP_001091521.1	Alpha-actinin-4	−2.06	0.0336
	XP_005209627.1	Dihydropyrimidinase-related protein 3 isoform X1	−1.92	0.0397
	NP_001263282.1	Lamin-B2	−1.72	0.0409
	NP_001030360.2	Transaldolase	−1.57	0.0319
	XP_005208438.1	Heterogeneous nuclear ribonucleoprotein H isoform X2	−1.42	0.0325
	XP_005222963.1	Filamin-B isoform X3	−1.00	0.0461
	NP_001029487.1	Adenosylhomocysteinase	−0.87	0.0509
	NP_776404.2	Actin, cytoplasmic 1	−0.80	0.0313
	XP_005226540.1	Vinculin isoform X3	−0.65	0.0276
	NP_776770.2	Heat shock cognate 71 kDa protein	−0.43	0.0184
	NP_001029421.1	Alcohol dehydrogenase class-3	0.84	0.0324
	XP_010814868.1	Growth factor receptor-bound protein 2 isoform X1	1.21	0.0144
	NP_777040.1	Superoxide dismutase (Cu-Zn)	1.36	0.0319
	XP_015328655.1	Talin-2	1.37	0.0387
	NP_001180044.1	Ras-related protein Rab-6A	1.41	0.0190
	XP_010805021.1	Peroxiredoxin-2 isoform X1	1.51	0.0311
	NP_001030358.1	Prostaglandin reductase 1	1.77	0.0180
	XP_010806674.1	Unconventional myosin-VI isoform X7	2.12	0.0115
	NP_776403.1	Low–molecular-weight phosphotyrosine protein phosphatase	2.14	0.0192
	NP_001103431.1	glutamine-fructose-6-phosphate aminotransferase (isomerising) 1	2.20	0.0420
	XP_005217265.1	Regulatory solute carrier protein family 1 member 1 isoform X1	2.27	0.0500
	XP_010806860.1	Band 4.1-like protein 2 isoform X4	2.57	0.0262
	NP_001091040.1	Biliverdin reductase A	2.98	0.0052
	NP_001070444.2	Uteroglobin precursor	4.31	0.0111
	NP_001095620.1	Phosphoserine aminotransferase	5.18	0.0201
Early Diapause – Elongated	NP_001028782.1	Keratin, type II cytoskeletal 8	−5.65	0.0414
	XP_002690791.3	Purine nucleoside phosphorylase	−5.21	0.0056
	NP_776393.1	Thioredoxin	−4.92	0.0427
	NP_776771.1	10 kDa heat shock protein, mitochondrial	−4.78	0.0044
	XP_005225065.1	Malate dehydrogenase, mitochondrial isoform X1	−4.75	0.0074
	NP_001068616.1	78 kDa glucose-regulated protein precursor	−4.68	0.0238
	NP_001179024.1	Keratin, type I cytoskeletal 18	−4.42	0.0228
	XP_015313986.1	60 kDa heat shock protein, mitochondrial isoform X1	−4.33	0.0283
	NP_001019738.1	Cystathionine gamma-lyase	−3.82	0.0117
	NP_001193589.1	Nucleolin	−3.67	0.0325
	XP_005203678.1	Lamin isoform X1	−3.63	0.0472
	NP_776993.1	Polyadenylate-binding protein 1	−3.22	0.0019
	NP_001178987.1	Annexin A10	−3.19	0.0258
	XP_005217320.1	Glutamine synthetase isoform X1	−3.18	0.0136
	XP_005220772.1	ATP-citrate synthase isoform X2	−3.16	0.0139
	NP_777125.1	Endoplasmin precursor	−3.10	0.0129
	XP_005211893.1	Kinectin isoform X5	−2.95	0.0128
	XP_002687260.2	Early endosome antigen 1 isoform X2	−2.95	0.0419
	XP_004008889.1	Stress-70 protein, mitochondrial isoform X1	−2.80	0.0421
	NP_001069978.2	Beta-hexosaminidase subunit beta preproprotein	−2.64	0.0413
	XP_012023387.1	Ribosome-binding protein 1 isoform X2	−2.42	0.0142
	XP_005214667.2	Microtubule-associated protein RP/EB family member 1 isoform X1	−2.40	0.0398
	XP_010811610.1	Guanine nucleotide-binding protein G(I)/G(S)/G(T) subunit beta-1 isoform X1	−2.38	0.0261
	XP_005219340.1	Protein arginine N-methyltransferase 1 isoform X1	−2.35	0.0011
	NP_777231.1	Aspartate aminotransferase, mitochondrial	−2.28	0.0487
	NP_991353.1	Staphylococcal nuclease domain-containing protein 1	−2.26	0.0400
	NP_786984.1	Isocitrate dehydrogenase [NADP], mitochondrial precursor	−2.21	0.0168
	NP_001263282.1	Lamin-B2	−2.20	0.0321
	NP_001095381.2	Protein RCC2	−2.04	0.0349
	NP_001091521.1	Alpha-actinin-4	−1.89	0.0125
	NP_001020505.1	Adenine phosphoribosyltransferase	−1.87	0.0365
	NP_001095811.1	Galectin-3	−1.74	0.0049
	NP_001030396.1	Ribonuclease inhibitor	−1.61	0.0342
	NP_001029666.1	Proliferating cell nuclear antigen	−1.56	0.0417
	XP_010806399.1	Heterogeneous nuclear ribonucleoprotein K isoform X2	−1.39	0.0279
	XP_005208438.1	Heterogeneous nuclear ribonucleoprotein H isoform X2	−1.33	0.0137
	NP_001015565.1	Poly(rC)-binding protein 1	−1.19	0.0058
	NP_001030360.2	Transaldolase	−1.18	0.0411
	NP_001029487.1	Adenosylhomocysteinase	−1.12	0.0477
	NP_776706.2	Coatomer subunit beta	−1.08	0.0500
	NP_001030524.1	Actin-related protein 2/3 complex subunit 5	−1.07	0.0319
	NP_776404.2	Actin, cytoplasmic 1	−1.05	0.0166
	NP_001029885.1	Actin-related protein 2/3 complex subunit 2	−0.97	0.0243
	XP_005222963.1	Filamin-B isoform X3	−0.96	0.0064
	XP_005226540.1	Vinculin isoform X3	−0.65	0.0071
	NP_776770.2	Heat shock cognate 71 kDa protein	−0.55	0.0318
	NP_001028795.1	Phosphatidylethanolamine-binding protein 1	0.85	0.0429
	XP_010815961.1	Microtubule-associated protein 4 isoform X10	1.06	0.0428
	NP_001073766.2	Switch-associated protein 70	1.15	0.0410
	XP_010813464.1	Echinoderm microtubule-associated protein-like 2 isoform X3	1.24	0.0144
	NP_001180044.1	Ras-related protein Rab-6A	1.25	0.0450
	XP_010814868.1	Growth factor receptor-bound protein 2 isoform X1	1.27	0.0073
	NP_001291895.1	Phosphoglucomutase-2	1.31	0.0386
	NP_777040.1	Superoxide dismutase (Cu-Zn)	1.36	0.0250
	XP_010805021.1	Peroxiredoxin-2 isoform X1	1.43	0.0304
	NP_001094668.1	Chromobox protein homolog 3	1.43	0.0472
	NP_001039517.1	Cytoplasmic dynein 1 light intermediate chain 1	1.44	0.0289
	NP_803470.2	Glycylpeptide N-tetradecanoyltransferase 1	1.53	0.0420
	NP_776866.1	26S protease regulatory subunit 8	1.56	0.0338
	NP_001178296.1	cAMP-dependent protein kinase type II-alpha regulatory subunit	1.58	0.0478
	XP_002686439.1	Protein FAM151A isoform X1	1.66	0.0354
	XP_010804133.2	Tetratricopeptide repeat protein 38 isoform X1	1.72	0.0426
	NP_001015647.1	60S ribosomal protein L10a	1.81	0.0426
	XP_015330819.1	Arfaptin-1 isoform X3	1.83	0.0308
	NP_776619.1	Tubulin-specific chaperone D	1.91	0.0303
	NP_001029871.1	Proliferation-associated protein 2G4	1.91	0.0032
	NP_001193081.1	Cytoplasmic dynein 1 light intermediate chain 2	1.92	0.0430
	NP_776403.1	Low-molecular-weight phosphotyrosine protein phosphatase	1.93	0.0472
	NP_001076083.1	UV excision repair protein RAD23 homolog A	1.95	0.0403
	NP_776850.1	1-phosphatidylinositol 4,5-bisphosphate phosphodiesterase gamma-1	1.97	0.0140
	NP_001039888.1	4-trimethylaminobutyraldehyde dehydrogenase	1.99	0.0396
	NP_001014855.1	26S protease regulatory subunit 4	2.02	0.0302
	XP_010803703.1	Branched-chain-amino-acid aminotransferase, cytosolic isoform X3	2.04	0.0427
	NP_001073091.1	Eukaryotic translation initiation factor 3 subunit G	2.07	0.0407
	XP_015330995.1	Density-regulated protein	2.11	0.0484
	NP_001019739.1	40S ribosomal protein S11	2.15	0.0228
	NP_001180149.1	ATP-dependent 6-phosphofructokinase, platelet type	2.15	0.0292
	NP_001029452.1	Leukotriene A-4 hydrolase	2.15	0.0252
	XP_002690482.2	Ras GTPase-activating-like protein IQGAP2 isoform X1	2.23	0.0412
	NP_001069351.1	Cytoplasmic dynein 1 intermediate chain 2	2.27	0.0239
	NP_001094683.1	Isochorismatase domain-containing protein 1	2.35	0.0254
	XP_005218560.1	IST1 homolog isoform X1	2.37	0.0044
	NP_001030358.1	Prostaglandin reductase 1	2.37	0.0062
	NP_001103431.1	glutamine-fructose-6-phosphate aminotransferase (isomerising) 1	2.42	0.0230
	NP_001040025.1	Glutathione S-transferase Mu 3	2.60	0.0502
	XP_010802603.1	Secernin-1 isoform X1	2.66	0.0384
	NP_001030548.1	Spermine synthase	2.78	0.0476
	NP_001039509.1	UMP-CMP kinase	2.85	0.0376
	XP_010806674.1	Unconventional myosin-VI isoform X7	2.90	0.0056
	NP_001091040.1	Biliverdin reductase A	3.10	0.0028
	XP_015316025.1	Stathmin isoform X1	3.43	0.0113
	NP_001015613.1	Creatine kinase B-type	3.72	0.0394
	NP_001070444.2	Uteroglobin precursor	3.88	0.0403
	NP_001095620.1	Phosphoserine aminotransferase	5.34	0.0133

A positive or negative log_2_ FC indicate higher abundance at the first or second developmental stage, respectively. Comparisons are as follows: Early – Late Diapause, Mid – Late Diapause, Late Diapause – Elongated, Mid-Diapause – Elongated and Early Diapause – Elongated.

**Figure 2:**
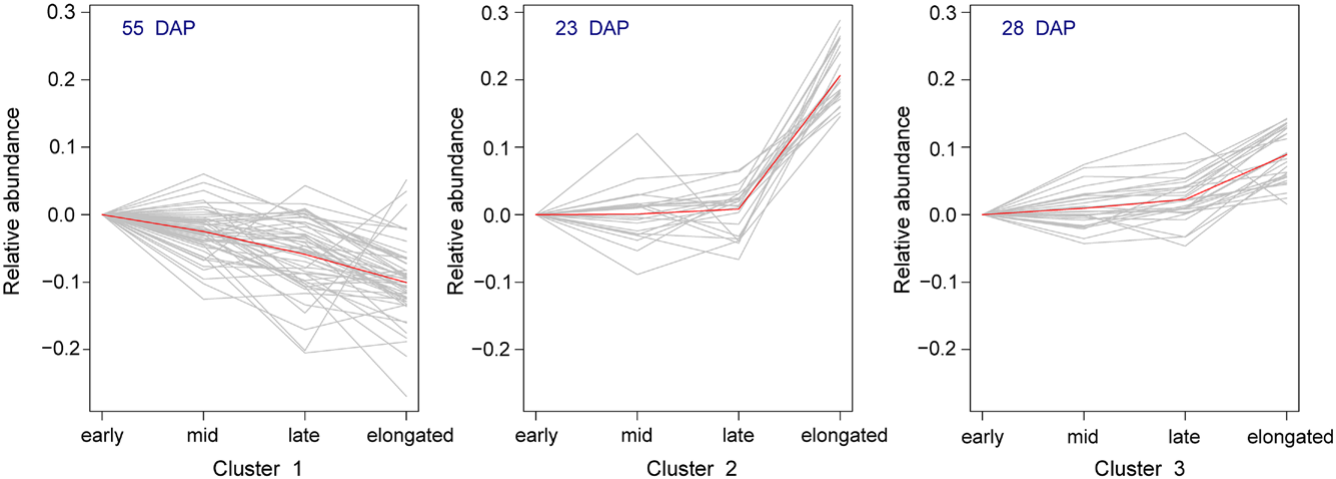
Self-organising tree algorithm on differentially abundant proteins reveals three clusters, namely clusters showing (1) a gradual decrease over development (55 DAP); (2) a stable abundance during the phases of diapause and sharp increase at elongation (23 DAP) and (3) a gradual increase over development (28 DAP).

To gain insight into the dynamic changes over development, a SOTA was used. The analysis revealed three clusters (Fig. 2), where the DAP showed one of the following abundance patterns: (1) a gradual decrease over development (55 proteins); (2) stable abundance during the phases of diapause and sharp increase at elongation (23 proteins); and (3) gradual increase over development (28 proteins).

### Biological functions

**Figure 3:**
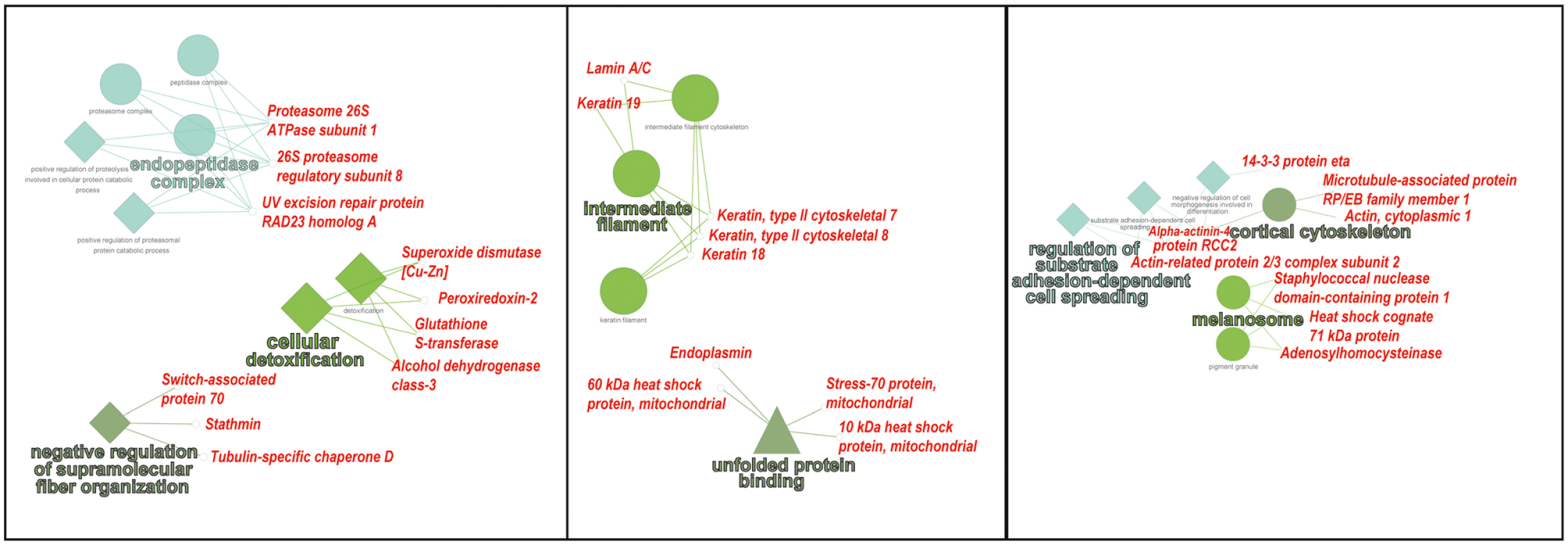
CluePedia of differentially abundant proteins in the three categories derived from the SOTA analysis. GO-BiologicalProcess, GO-CellularProcess and GO-MolecularFunction are displayed by a square, circle and triangle, respectively. The proteins are displayed in *red* and the GO terms are displayed in coloured text.

The proteins that were not statistically significantly different between the developmental stages were subjected to GO to identify enriched pathways in proteins that support embryo development during a prolonged period of decelerated embryo development. The top five enriched clusters were GO:0070062~extracellular exosome, GO:0005912~adherens junction, GO:0005913~cell-cell adherens junction, GO:0000502~proteasome complex, and GO:0044445~cytosolic part. A full overview of all enriched clusters can be found in Supplementary Table 2. The biological function of the differentially expressed proteins in the three SOTA-derived clusters was further analysed by subjecting the DAP to ClueGO and CluePedia to visualise the functional interactions (Table 2 and Fig. 3). Proteins in cluster 1, where the relative abundancy of proteins decreased over embryo development, were in the GO categories ‘endopeptidase complex’ (GO:1905369), ‘cellular detoxification’ (GO:1990748) and ‘negative regulation of supramolecular fibre organisation’ (GO:1902904). Proteins in cluster 2, where the abundance is stable during diapause and increase at elongation, were in GO categories ‘intermediate filament’ (GO:0005882) and ‘unfolded protein binding’ (GO:0051082). Lastly, proteins in cluster 3, where protein abundance increases over development, were in GO categories ‘cortical cytoskeleton’ (GO:0030863), ‘regulation of substrate adhesion-dependent cell spreading’ (GO:1900024) and ‘melanosome’ (GO:0042470).

**Table 2:** Gene ontology of differentially abundant proteins in cluster 1, 2 and 3 derived from the SOTA analysis.

Cluster	GO category	GO term	Proteins
Cluster 1	Endopeptidase complex	GO:1905369	Proteasome 26S ATPase subunit 1
			26S proteasome regulatory subunit 8
			UV excision repair protein RAD23 homolog A
	Cellular detoxification	GO:1990748	Alcohol dehydrogenase class-3
			Glutathione S-transferase
			Peroxiredoxin-2
			Superoxide dismutase [Cu-Zn]
	Negative regulation of supramolecular fibre organisation	GO:1902904	Stathmin
			Switch-associated protein 70
			Tubulin-specific chaperone D
Cluster 2	Intermediate filament	GO:0005882	Keratin 18
			Keratin 19
			Keratin, type II cytoskeletal 7
			Keratin, type II cytoskeletal 8
			Lamin A/C
	Unfolded protein binding	GO:0051082	Endoplasmin
			10 kDa heat shock protein, mitochondrial
			Stress-70 protein, mitochondrial
			60 kDa heat shock protein, mitochondrial
Cluster 3	Cortical cytoskeleton	GO:0030863	Actin, cytoplasmic 1
			Alpha-actinin-4
			Microtubule-associated protein RP/EB family member 1
	Regulation of substrate adhesion-dependent cell spreading	GO:1900024	Alpha-actinin-4
			Actin-related protein 2/3 complex subunit 2
			protein RCC2
	Melanosome	GO:0042470	Adenosyl homocysteinase
			Heat shock cognate 71 kDa protein
			Staphylococcal nuclease domain-containing protein 1

### Specific proteins related to diapause and reactivated embryo development

Several DAPs were specifically related to embryonic growth repression and reactivation of embryo development. In cluster 1, there was a high abundance of 4-trimethylaminobutyraldehyde dehydrogenase (ALDH9A1) and spermine synthase (SMS) during early diapause, which were significantly lower abundant during elongation. Both proteins have been implicated in the amine and polyamine biosynthesis pathway. In cluster 2, several proteins including ATP-citrate synthase (ACLY), nucleolin (NCL), lamin A/C (LMNA) and purine phosphorylase (PNP), which are involved in cell proliferation, were higher abundant at elongation than during diapause. Lastly, in cluster 3, protein arginine N-methyltransferase 1 (PRMT1), heterogeneous nuclear ribonucleoprotein K (HNRNPK), aspartate aminotransferase (AST) and tissue alpha-L-fucosidase (FUCA1) were higher abundant at elongation than during diapause.

## Discussion

The uterine environment notably changes and adapts to the embryos’ needs to support early development and survival. There was only a limited overlap of the DAP in the present study with DAP reported in other species analysed so far. As field sampling does not allow adequate determination of the day of pregnancy, a direct interspecies comparison was not conducted. Data from different published studies in various species was used to gain insight into potentially important factors for the regulation of diapause and reactivation of embryo development in the roe deer. The total number of 819 identified and quantified uterine fluid proteins falls within the observed range (between 299 and 1359) in various species including cows, mouse, pig and horse (Kayser *et al.* 2006, Mullen *et al.* 2012, Forde *et al.* 2014, 2015, Jalali *et al.* 2015, Kelleher *et al.* 2016, Martin *et al.* 2016, Smits *et al.* 2018).

We hypothesised to find different abundance profiles, including a gradual increase or decrease during pregnancy, as well as a stable abundance during diapause followed by a sharp increase or decrease at the onset of elongation. In line with the lack of embryo elongation in the uterine gland knockout sheep model (Gray *et al.* 2002), most DAP in this study were identified in the uterine fluid between early diapause and elongation. Surprisingly, a cluster with stable abundance during diapause, but sharp decrease at elongation did not appear in the present dataset. This potentially indicates an active reactivation rather than an active repression of embryo development.

GO was used to gain insight into the functions of the proteins in the three different clusters. In cluster 1, ‘endopeptidase complex’, ‘cellular detoxification’, and ‘negative regulation of supramolecular fibre organisation’ were represented. The proteins in this clusters showed a significantly lower abundance at elongation than during diapause. Proteins in the ‘endopeptidase complex’ are involved in breaking peptide bonds within proteins. Proteins involved in ‘cellular detoxification’ are recruited to remove toxic substances, whereas proteins involved in ‘negative regulation of supramolecular fibre organisation’ are involved in processes that stop, prevent or reduce the frequency, rate or extent of fibril organisation. None of the proteins representing the ‘endopeptidase complex’ was quantified in cattle on day 10, 13, 16 and 19 of pregnancy (Forde *et al.* 2014). The proteins alcohol dehydrogenase class-3, glutathione S-transferase and peroxiredoxin-2 in the category ‘cellular detoxification’ and the protein stathmin in the category ‘negative regulation of supramolecular fibre organisation’ decreased like in day 10 to 19 pregnancy in cattle (Forde *et al.* 2014). In addition, alcohol dehydrogenase class-3 and superoxide dismutase (Cu-Zn) showed a non-significant but gradual decrease from day 3 to day 4 of pregnancy in mice (Kelleher *et al.* 2016), indicating a species-independent gradual decrease of proteins involved in cellular detoxification before implantation. Moreover, the abundance of peroxiredoxin-2 was significantly higher in pregnant versus cyclic cattle on day 8 of pregnancy (Munoz *et al.* 2012), the abundance of glutathione S-transferase was significantly higher in pregnant versus cyclic horses on day 13 (Smits *et al.* 2018), and the abundance of superoxide dismutase (Cu-Zn) increased in uterine fluid from cycle day 10 to 13 in cattle (Mullen *et al.* 2012). This highlights that these proteins are altered by the presence of an embryo and thus are likely to play an important role during early pregnancy.

In cluster 2, ‘intermediate filament’ and ‘unfolded protein binding’ were represented. The proteins in this clusters showed a sharp increase at elongation and were significantly higher abundant at elongation than during diapause. The proteins in ‘intermediate filament’ are involved in the cytoskeletal structure in eukaryotic cells and keratin 18, keratin, type II cytoskeletal 7, keratin, type II cytoskeletal 8, but not keratin 19 and lamin A/C showed an increased abundance at elongation like in cattle (Forde *et al.* 2014). All identified keratin proteins consisted of at least one roe deer specific peptide. In the porcine endometrial tissue, the abundance of keratin, type II cytoskeletal 8 was significantly decreased on day 12 versus day 9 of pregnancy (Kayser *et al.* 2006). A similar abundance pattern was found in mice for keratin 19, which was high on day 3, significantly lower on day 4, but not different between day 4 and 5 in mice (Kelleher *et al.* 2016). Nevertheless, keratin 19 abundance seems important for ruminant pregnancy, as it was significantly more abundant in uterine fluid of pregnant versus cyclic cattle on day 8 (Munoz *et al.* 2012). The proteins involved in ‘unfolded protein binding’ are interacting selectively and non-covalently with an unfolded protein, but none of the proteins in that category followed the relatively stable abundance as observed in bovine (Forde *et al.* 2014). However, it has been shown that the 10 kDa heat shock protein, mitochondrial is produced by the bovine embryo and can be detected in uterine fluid from day 16 onwards, which is in line with the sharp increase in abundance at elongation in the roe deer uterine fluid (Forde *et al.* 2015).

In cluster 3, proteins involved in ‘cortical cytoskeleton’, ‘regulation of substrate adhesion-dependent cell spreading’ and ‘melanosome’ were represented. The proteins in these clusters showed a significantly higher abundance at elongation than during diapause. The proteins in ‘cortical cytoskeleton’ are proteins that lie just beneath the plasma membrane and actin, cytoplasmic 1, but not alpha-actinin-4, and microtubule-associated protein RP/EB family member 1 showed a gradual increase in cattle like observed in roe deer (Forde *et al.* 2014). Like in roe deer, actin, cytoplasmic 1 significantly increased from day 9 to day 12 of pregnancy in pigs (Kayser *et al.* 2006). The proteins in ‘regulation of substrate adhesion-dependent cell spreading’ are proteins that are involved in any process that modulates the frequency, rate or extent of substrate adhesion-dependent cell spreading. Lastly, proteins in the category ‘melanosome’, which is a membrane-bound cytoplasmic organelle within which melanin pigments are synthesised and stored, did not follow the gradual increase observed in roe deer in either cattle or mice (Forde *et al.* 2014, Kelleher *et al.* 2016).

The function of individual proteins that were not categorised by GO were assessed to gain insight into their potential role in the regulation of growth repression and/or reactivation of embryo development. The proteins 4-trimethylaminobutyraldehyde dehydrogenase (ALDH9A1) and spermine synthase have been implicated in the amine and polyamine biosynthesis. In roe deer, both proteins decreased in abundance at elongation compared to diapause. In cattle, ALDH9A1 decreased from day 10 to 13 of pregnancy and stayed fairly stable from day 16 to day 19 of pregnancy (Forde *et al.* 2014). The spermine synthase decreased both from day 10 to 13 and day 16 to 19 of pregnancy in cattle (Forde *et al.* 2014) and decreased from day 9 to day 12 of pregnancy in the porcine endometrium (Jalali *et al.* 2015). In light of diapause regulation, the presence of polyamines is particularly interesting, as inhibition of polyamine synthesis causes entry of the mouse (Fenelon & Murphy 2017), hamster (Galliani *et al.* 1983) and rat (Reddy & Rukmini 1981) blastocyst into embryonic diapause. An increase in putrescine, a polyamine, has been found to be increased at reactivation (Lefevre *et al.* 2011) and can even cause reactivation of embryo development in mink blastocysts (Fenelon *et al.* 2016). Ornithine decarboxylase (ODC1), which converts ornithine into polyamine, has a higher abundance in endometrial luminal, glandular epithelium and subepithelial stroma upon embryo reactivation in mink (Lefevre *et al.* 2011). ODC1 was not identified in the uterine fluid of roe deer, but polyamine synthesis could be rescued by the arginine-agmatine-putrescine (ADC/AGMAT-dependent) pathway, as has previously been confirmed to be the compensatory pathway in ODC1-knockdown sheep embryos (Wang *et al.* 2014). Spermine synthase catalyses the conversion of spermidine into spermine (both metabolites of putrescine) and ALDH9A1 catalyses the conversion of 4-aminobutanal (a putrescine metabolite) into GABA (Ippolito & Piwnica-Worms 2014). The role of polyamines in the regulation of diapause in roe deer remains speculative. However, putrescine is one of the main drivers of cell proliferation (Fenelon *et al.* 2016) and can thereby cause embryo reactivation after embryonic diapause. The reduced expression of ALDH9A1 and spermine synthase in roe deer could imply an accumulation of putrescine at the end of diapause, which promotes reactivation of embryo development.

In line with the reactivation of embryo development, proteins involved in cell proliferation including ATP-citrate synthase, nucleolin, lamin A/C and purine phosphorylase were higher abundant at elongation than during diapause in the roe deer. The ATP-citrate synthase, which is the enzyme that synthesises cytosolic acetyl CoA, did not increase at elongation in cattle as observed in roe deer (Forde *et al.* 2014). However, acetyl CoA has been shown to increase after blastulation in *Xenopus laevis* and is known to play a role in the regulation of cell proliferation by affecting protein acetylation reactions (Tsuchiya *et al.* 2014). Nucleolin abundance stayed stable in mice (Kelleher *et al.* 2016), but has been implicated to play a role in progression through the S-phase of the cell cycle and is thus important for cell proliferation (Xiao *et al.* 2014). Although important for stem cell differentiation (Sehgal *et al.* 2013), lamin A/C decreased at elongation in cattle (Forde *et al.* 2014), whereas it sharply increased at elongation in the roe deer. Lastly, purine nucleoside phosphorylase, an enzyme involved in metabolism of nucleotides with stimulatory effects on cell proliferation (Boehncke *et al.* 1994) decreased from day 10 to 13 and day 16 to 19 of pregnancy in cattle (Forde *et al.* 2014).

Proteins with a gradual increase during diapause and elongation included arginine N-methyltransferase 1, heterogeneous nuclear ribonucleoprotein K, aspartate aminotransferase and tissue alpha-L-fucosidase. These proteins play a role in amino acid homeostasis, DNA damage repair and fructose synthesis and are thus important for cell proliferation (Pawlak *et al.* 2000, Ezawa *et al.* 2016, Zhang *et al.* 2016, Feist *et al.* 2018). In cattle, pigs or mice, none of these proteins has been reported to show a rise prior to implantation as observed in roe deer. Supported by the findings in tammar wallaby (Martin *et al.* 2016), roe deer embryo development may be reactivated by the presence of positive regulators of the cell cycle. In the tammar wallaby, the high abundance of cell cycle inhibition proteins during diapause switches to increased abundance of growth factors once the embryo resumes development (Martin *et al.* 2016).

We provide evidence that the abundance of the proteins in the uterine environment of the pre-implantation roe deer embryo remain stable during the period of diapause, while changes occur upon embryo elongation. The observed pattern of DAP, concurrent with the morphological change from the diapausing blastocyst to the elongated embryo, indicates that the uterine fluid proteome during diapause has a high antioxidant capacity and that the embryo is kept in diapause in the absence of polyamines. In addition, embryo development may be actively resumed through the presence of cell cycle promoters at elongation.

## Supplementary Material

Supplementary Table 1. LFQ of all identified proteins

Supplementary Table 2. DAVID cluster analysis of proteins with stable abundance

## Declaration of interest

The authors declare that there is no conflict of interest that could be perceived as prejudicing the impartiality of the research reported.

## Funding

The study was funded by the Swiss National Science Foundation SNSF (159734).

## Author contribution statement

V A v d W performed sample collection, coordinated the experiments, analysed the data and wrote the manuscript. J T B performed the proteome assembly. S B supervised the proteome assembly. T F and G A conducted the proteome experiment and supervised the data analysis. B D rendered possible and coordinated field sampling and performed sample collection. S E U conceptualised the study, coordinated and supervised the project and revised the manuscript. All authors read, edited and approved the final manuscript.
